# Sulphide Re-Os geochronology links orogenesis, salt and Cu-Co ores in the Central African Copperbelt

**DOI:** 10.1038/s41598-018-33399-7

**Published:** 2018-10-08

**Authors:** N. J. Saintilan, D. Selby, R. A. Creaser, S. Dewaele

**Affiliations:** 1 Department of Earth Sciences, University of Durham, Durham, DH1 3LE United Kingdom; 2 State Key Laboratory of Geological Processes and Mineral Resources, School of Earth Resources, China University of Geosciences, Wuhan, China; 3 Department of Earth and Atmospheric Sciences, University of Alberta, Edmonton, Alberta T6G 2E3 Canada; 4 Royal Museum for Central Africa, Leuvensesteenweg 13, B-3080 Tervuren, Belgium; 5 Mineralogy and Petrology, Department of Geology, Ghent University, Krijgslaan 281 S8, B-9000 Ghent, Belgium; 6 Present Address: Institute of Geochemistry and Petrology, Department of Earth Sciences, ETH Zürich, Clausiusstrasse 25, 8092 Zürich, Switzerland

## Abstract

The origin of giant, sedimentary rock-hosted copper-cobalt (Cu-Co) provinces remains contentious, in part due to the lack of precise and reliable ages for mineralisation. As such, no consensus has been reached on the genetic model for ore formation, and the relationships between tectonism, palaeo-fluid circulation and mineralisation. Here, we link the timing of Cu-Co mineralisation in the Central African Copperbelt to compressional tectonics during the Lufilian Orogeny by using new ca. 609–473 Ma ages given by rhenium-osmium (Re-Os) isotope data for individual Cu-Co sulphides (carrolite and bornite) from the Cu-Co Kamoto deposit. The initial Os isotope composition of carrolite is compatible with the leaching of Os and Cu(-Co) from Mesoproterozoic Cu sulphide deposits hosted in fertile basement. In contrast, the ca. 473 Ma Cu-Au mineralisation stage, which is coeval with late- to post-compressional deformation, may be a distal expression of fluid flow and heat transfer caused by magmatic intrusions in the core of the collisional orogen. The Re-Os ages support a model for mineralisation driven by evaporite dissolution and percolation of large volumes of dense brines in the Katangan Basin during the Lufilian Orogeny.

## Introduction

**Figure 1:**
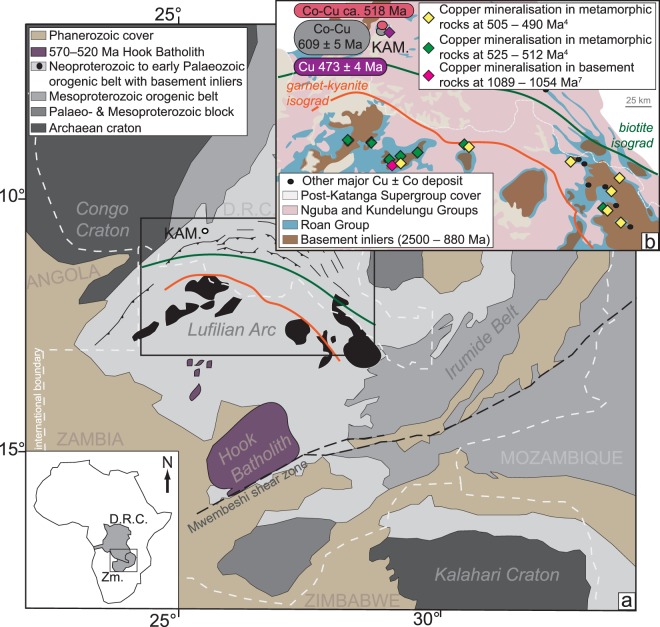
(**a**) Location of the Lufilian Arc and Hook batholith in Zambia and the Democratic Republic of Congo with respect to the Archaean Kalahari and Congo cratons and the Mesoproterozoic orogenic belts (after ref.^24^). Acknowledgement: The base map was reprinted by permission from “Ore Geology Reviews, vol. 35, Kampunzu, A.B., *et al*., Sediment-hosted Zn-Pb-Cu deposits in the Central African Copperbelt, p. 263–297, Copyright (2009), with permission from Elsevier”. The metamorphic isograds were reprinted from “Ore Geology Reviews, vol. 54, Eglinger, A. *et al*., Geochemical signatures of uranium oxides in the Lufilian belt: From unconformity-related to syn-metamorphic uranium deposits during the Pan-African orogenic style, p. 197–213, Copyright (2013), with permission from Elsevier”. (**b**) Major copper-cobalt (Cu-Co) deposits in the Central African Copperbelt in the Lufilian Arc. The deposits are classified according to the Re-Os ages of molybdenite associated with Cu mineralisation in metasedimentary or granite basement host rocks (after refs^4,8,42^). The metamorphic isograds are after ref.^43^. Abbreviation: Kam.: Kamoto. Acknowledgements: The base map was reprinted by permission from “Springer Nature: Mineralium Deposita, vol. 52, Age of the Zambian Copperbelt, Sillitoe, R.H., Perelló, J., Creaser, R.A., *et al*., p. 1245–1268, Copyright, 2017”. The metamorphic isograds were reprinted from “Ore Geology Reviews, vol. 54, Eglinger, A. *et al*., Geochemical signatures of uranium oxides in the Lufilian belt: From unconformity-related to syn-metamorphic uranium deposits during the Pan-African orogenic style, p. 197–213, Copyright (2013), with permission from Elsevier”.

The most important source of copper (Cu), beyond that of giant and supergiant porphyry Cu deposits, is sedimentary rock-hosted stratiform and vein-type Cu deposits. Mining of economical mineralisation is centred on the Udokan deposit in the Palaeoproterozoic Kodaro-Udokan Basin in Siberia, Russian Federation^1^, the Central African Copperbelt in the Neoproterozoic Katangan Basin in Zambia and the Democratic Republic of Congo^2^ (DRC, Fig. 1a) and the Permian Zechstein Basin of Central Europe^3^. Containing ca. 200 Mt of Cu and a significant supply of cobalt (Co), the Central African Copperbelt along the Lufilian fold-and-thrust belt (i.e., Lufilian Arc, Fig. 1a) is the world’s most important metallogenic province of this kind^4^.

Recent uranium-lead (U-Pb) and rhenium-osmium (Re-Os) geochronological studies have revealed that several major sediment-hosted stratiform and vein-type Cu deposits formed during orogenesis and the associated metamorphism (e.g., Udokan and Nussir in Norway)^1,5^, or mineralisation coincided with the timing of basin-wide intersecting folding of basinal strata (e.g., Dzhezkazgan in Kazhakstan)^6^. In parallel, Re-Os ages of molybdenite, which is paragenetically associated with Cu-sulphides, were combined with previous structural, mineralogical and geochemical evidence in several ore deposits in the Zambian part of the Central African Copperbelt, where the host rocks were metamorphosed to the greenschist to amphibolite facies^4,7,8^ (Fig. 1b). An epigenetic introduction of Cu as stratiform and veinlet-type sulphide mineralisation during the peak and post-peak stages of the Lufilian collisional orogeny in those Zambian deposits was proposed (540–490 Ma)^4,7–10^. Although the most effective time for fluid mobilization, overpressuring, and expulsion of metal-bearing brines is related to contraction and fault inversion^4,11,12^, this genetic model was criticised^13,14^ in favour of the popular paradigm of older, primary syndiagenetic Cu introduction followed by possible orogenic overprint/remobilization^2,15–18^.

To address this controversy, we present new Re-Os isotope geochemistry and geochronology data from mineral separates of individual sulphide species (i.e., carrolite–CuCo_2_S_4_, bornite–Cu_5_FeS_4_) from fifteen mineralised samples from the sedimentary horizons comprising the Upper and Lower Orebodies at the Cu-Co Kamoto deposit, in the western part of the Central African Copperbelt, Katanga province, DRC (see Supplementary Data Table for the lithostratigraphic positions of these samples). Unlike those Cu-dominated deposits in Zambia, the sedimentary rocks hosting the Kamoto deposit represent some of the least deformed host rocks in the Central African Copperbelt, and are only weakly metamorphosed with growth of white mica and chlorite during burial and compressional tectonics of the Lufilian Orogeny^15,17^ (Fig. 1b). Together with our new Re-Os ages, underpinned by new petrographical data, we reinterpret previous fluid inclusion microthermometry and radiogenic strontium (^87^Sr/^86^Sr) isotope data from gangue minerals (i.e., quartz and dolomite) associated with carrolite and bornite at Kamoto^15,19^. The robust Re-Os ages presented here clearly place all stages of ore mineralisation studied at Kamoto into an orogenic framework, not associated with an early burial-diagenetic model. Additionally, we discuss the possible roles of the demise of mid-Neoproterozoic ice ages followed by the building of the Lufilian fold-and-thrust belt with known linkages to salt tectonics, for the formation of the giant, sedimentary rock-hosted Cu-Co ores of the Central African Copperbelt^20^ based on the new geochronological data.

### Evaporite breccias, salt tectonics, and the origin of brines

**Figure 2:**
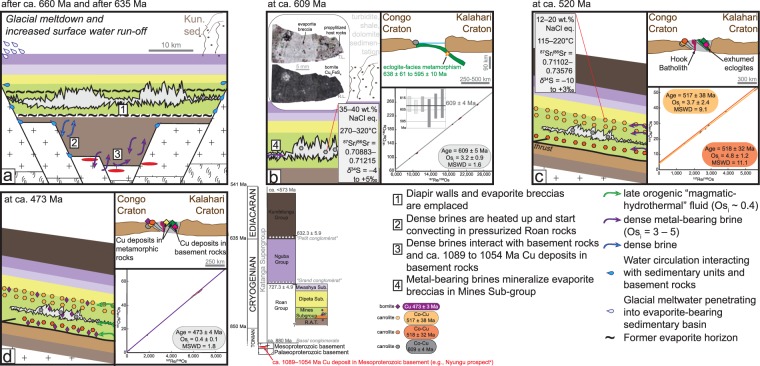
Tectonic scenario for the Neoproterozoic sedimentary rocks of the Katanga basin (**a**) after ca. 660 Ma, (**b**) at ca. 609 Ma, (**c**) at ca. 520 Ma, (**d**) at ca. 473. Ma. The dynamics of fluid circulation and the fluids characteristics of each stage of Cu-Co mineralisation are explained in each case. The dynamics of formation of the evaporitic breccias and the origin of dense saline brines are explained after ref.^23^. In addition, each mineralisation stage defined by its Re-Os age for Cu-Co or Cu mineralisation is placed in the framework of Lufilian Orogeny from late basin sedimentation to basin inversion and final continent-continent collision (**a**–**c**), prior to orogenic uplift and cooling (**d**). Individual ellipses show the error situation of each data point in ^187^Os/^188^Os vs. ^187^Re/^188^Os space (i.e., Re-Os isochron diagrams). Ellipses are constructed from the maximum and minimum error vectors that are orthogonal to one another. Maximum and minimum errors are statistical values that are calculated from the uncertainty of the ^187^Os/^188^Os and ^187^Re/^188^Os ratios for a given data point. Final uncertainties were calculated by full error propagation of uncertainties in the Re and Os measurements, blank values, isotopic compositions, spike calibrations, and reproducibility of the standard Re and Os values. The error correlation function rho is utilized for isochron regressions. The uncertainty in the ^187^Re decay constant is included in the isochron and model ages uncertainty (refs^52,56^). Abbreviation: Kun. Sed.: Sedimentation of Kundelungu Group.

The Central African Copperbelt is hosted by metasedimentary siliciclastic and carbonate rocks of the Roan Group (ca. 880–727 Ma) that comprise the lower part of the Katanga Supergroup deposited in the Neoproterozoic Katangan Basin between ca. 880 and ca. <573 Ma (stratigraphy in legend of Fig. 2)^21–24^. The Roan Group low-energy sedimentary units are characterised by dissolution relics of evaporites (e.g., remnant sabkha facies, gypsum and anhydrite pseudomorphs, collapse breccia and stratigraphic gaps^21–23^; Fig. 2a,b), cross-cutting evaporitic megabreccias and salt diapirs extending into the Nguba (ca. 727–632 Ma) and Kundelungu (ca. 632– < 573 Ma) Groups, and propylitized host rocks on the edges of evaporite megabreccias^23,24^ (Fig. 2a,b).

**Figure 3:**
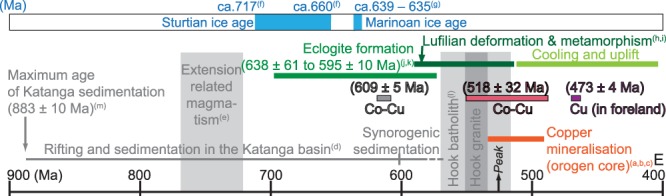
Time chart (modified after ref.^4^) of the ages of Cu-Co mineralisation in the Congolese Copperbelt (this study) and of Cu ± Co mineralisation in the metamorphosed sedimentary rocks in the Zambian Copperbelt (ref. (**a**) ref.^42^; refs (**b**), (**c**) refs^4,8^) relative to Katangan sedimentation (ref. (**d**) ref.^2^), extension-related magmatism (ref. (**e**) ref.^17^), Sturtian and Marinoan ice ages (refs (**f**,**g**) refs^25,31^), Lufilian deformation and metamorphism, including post-orogenic uplift and cooling (ref. (**h**) ref.^35^; ref. (**i**) ref.^24^), eclogite-facies metamorphism and orogenic peak (refs (**j**,**k**) refs^41,57^), Hook batholith emplacement (ref. (**l**) ref.^45^). The maximum age for Katangan sedimentation is based on the U-Pb zircon age of the Nchanga basement granite (ref. (**m**) ref.^58^).

The six aliquots of carrolite, which replace evaporitic breccias in the Mines Sub-group at Kamoto (see Supplementary Figure 1), yield a Model 1 Re-Os isochron age of 609 ± 5 Ma and a weighted average of the model ages of 609 ± 4 Ma (using the isochron initial ^187^Os/^188^Os ratio [Os_i_] that corresponds to the isotopic composition of common Os incorporated at the time of sulphide precipitation – Os_i_ = 3.2 ± 0.9, Fig. 2b). These concordant ages not only date Co-Cu mineralisation, but also place a minimum age limit for the building of evaporitic breccia and salt diapir tectonism at the transition from basin sedimentation to syn-orogenic sedimentation during deposition of the Kundelungu Group (Figs 2 and 3).

The restored pre-dissolution stratigraphy of the 1000 m-thick Roan Group shows four, 150- to 500-m-thick evaporite horizons^23^. In mid-Roan time, during sedimentation, small salt walls and extrusion of evaporite breccia began to be passively emplaced^23^. During sedimentation of the Nguba and Kundelungu Groups (ca. 727 to after 632 Ma)^23–25^, enlarged evaporitic diapirs continued to be passively emplaced while siliciclastic sediments accumulated around and above them^22^. The break-up of ice sheets and the dramatic volumes of water, released during deglaciation after the Cryogenian snowball episodes (Figs 2a and 3, after ca. 660 Ma and after ca. 635 Ma, respectively^25–31^), triggered the rise in global mean sea level and an increased run-off of surface waters. These palaeo-environmental conditions may have led to enhanced sedimentation of siliciclastic sediments in the region of the Katangan Basin with accumulation of siliciclastic sediments of the Kundelungu Group after ca. 632 Ma which would have caused compaction and bolstered salt tectonics^23,25^.

The melt-down of Marinoan ice sheets after ca. 635 Ma, which resulted in surface waters dominated by glacial meltwaters from which well-developed transgressive cap dolostones were deposited^25–31^, may have favoured the dissolution of the evaporite horizons in the Roan Group^20,32^ (Fig. 2a). Indeed, the penetration of glacial meltwaters into sedimentary basins to depths of ca. 1000 m can disrupt relatively stagnant fluids and create a strong disequilibrium pattern in fluid salinity (e.g., the impacts of Pleistocene glaciation in the Michigan Basin^33,34^). This disequilibrium could have favoured the dissolution of the thick evaporite horizons in the Roan Group in order to maintain high salinities in the Katangan Basin^20,32,33^. Therefore, large volumes of dense saline brines may have accumulated in the Roan Group penecontemporaneously with siliciclastic sedimentation of the Kundelungu Group that applied continued compression and burial onto the underlying Roan Group^23^ (Fig. 2a). Then, the dense brines would have been heated and started convecting in the pressurized Roan Group^22^ for ca. 20 Myr. from ca. 632 Ma to at least ca. 609 Ma, coinciding with the initial phases of the Lufilian Orogeny^24,35^ and the earliest type of Cu-Co mineralisation at Kamoto.

### Pre-enriched Mesoproterozoic basement and source(s) of Cu and Co

Large-scale, low-velocity convection of high salinity aqueous fluids in sedimentary basins has been demonstrated by numerical modelling considering the thermodynamic properties of brines^36^. In addition, dense basinal brines, which form an interconnected fluid network at a lower porosity than pure water^37,38^, even in the absence of cross-strata conduits^36^, eventually descend and involve fluid circulation in basement rocks as they reach the sedimentary-basement interface^36^ (Fig. 2a). The highly radiogenic Os_i_ of 3.2 ± 0.9 in epigenetic carrolite in the evaporite breccia, combined with the radiogenic ^87^Sr/^86^Sr compositions of 0.708–0.712 in coarse-grained dolomite associated with carrolite in the evaporite breccia^15^, strongly favours our hypothesis that dense brines interacted with Mesoproterozoic basement rocks containing a source of radiogenic Os and Sr, such as the ca. 1089 to 1054 Ma Cu deposits that were formed during the Irumide collisional orogeny (e.g., Nyungu prospect, Re-Os molybdenite ages^8^). These hot (270–320 °C) and dense brines with salinity of 35–40 wt.% NaCl eq. were suitable media for the transport of Cu in solution as chloride complexes^39^ (microthermometry data in quartz associated with carrolite in evaporite breccia^19^). In addition, Co, the solubility of which increases with Cl content of hydrothermal fluids, is primarily transported as CoCl_4_^2−^ in such fluids^40^. Although Co may be a subsidiary component in those Cu deposits in Mesoproterozoic basement^7^, it is possible that Co was derived from Neoproterozoic eclogite, gabbro and metagabbro. Although such rocks have not been reported to date in basement rocks in the Congolese Copperbelt, this basement could bear similar rocks as those eclogites, gabbros and metagabbros presently found in central Zambia^41^. The geochemistry of these rocks and the 638 ± 61 Ma to 595 ± 10 Ma eclogite-facies metamorphism attest to subduction of Neoproterozoic oceanic crust to a depth of ca. 90 km in a cold Phanerozoic-like subduction zone associated with early Lufilian tectonics^41^ (Fig. 2b).

### Impact of protracted orogenic activity and metamorphism on metal distribution

Carrolite mineralisation in the Upper and Lower Orebodies yields indistinguishable Model 3 Re-Os isochron ages of 517 ± 38 Ma and 518 ± 32 Ma, respectively (Fig. 2c). These ages overlap with the peak stage of Lufilian metamorphism and continent-continent collision between the Congo and Kalahari Cratons^24,35^ when Roan Group sedimentary rocks were translated into a foreland setting (presently in DRC) within large-scale far-transported thrust sheets, using salt as lubricant^23^. The interconnected fluid network of dense and metal-bearing brines led to mineralisation in permeable units above and below the seal of evaporite breccias that had been mineralised at ca. 609 Ma. The Os_i_ ratios of stratiform carrolite mineralisation in the Upper and Lower Orebodies (3.7 ± 2.4 & 4.8 ± 1.2, respectively) overlap within uncertainty with the basement-sourced Os_i_ ratio of carrolite mineralisation in the evaporite breccia. However, the lower salinities (12–20 wt.% NaCl eq., microthermometry data in quartz associated with carrolite) and temperature (115–220 °C) of the hydrothermal fluids for stratiform carrolite mineralisation^19^, together with the more radiogenic ^87^Sr/^86^Sr ratios in fine-grained dolomite (0.711–0.735)^15^, may reflect the prolonged interaction of the original hydrothermal fluids with arenitic- and shale-type Roan Group sedimentary units, as well as, their coeval cooling and dilution by pore waters in the thrust sheets. This ca. 518–517 Ma stratiform carrolite mineralisation stage at Kamoto in a foreland setting during the Lufilian Orogeny is broadly coeval with the ca. 525–512 Ma stratiform and vein-type Cu ± Co deposits hosted by mostly garnet-kyanite isograd amphibolite-facies rocks in the core of the orogen (i.e., the Domes region in the Zambian part of the Central African Copperbelt)^4,42,43^.

In agreement with previous petrographic interpretations^15,19^, we identify that bornite precipitation post-dated carrolite mineralisation and, in places, bornite replaced carrolite. This extensive bornite mineralisation preceded the latest hypogene sulphide stage represented by chalcocite precipitation^15,19^ (see Supplementary Figure 1). Bornite, which is common to the Upper and Lower Orebodies and the evaporite breccia at Kamoto in foreland setting (Fig. 2d), formed at 473 ± 4 Ma (Model 1 Re-Os isochron age). Therefore, this bornite mineralisation stage coincides with the timing of orogenic uplift and cooling at ca. 512–470 Ma^35,44^ (Fig. 3), and formed ca. 20 Myr. after stratiform and vein-type Cu ± Co mineralisation that occurred in biotite isograd greenschist-facies rocks at ca. 505–490 Ma (i.e., the Domes region in the Zambian part of the Central African Copperbelt)^4,43^.

The bornite Os_i_ of 0.4 ± 0.1 precludes the Mesoproterozoic basement including those Irumide-time Cu deposits from being the source of the common Os. Previous studies identified the possibility of a bornite stage that preceded and was locally replaced by carrolite at Kamoto^15^. In fact, if our ca. 473 Ma bornite stage, which is common to the evaporite breccia and stratiform mineralisation styles, resulted from the dissolution/reprecipitation of an older bornite (or other Cu-Co sulphide) stage, we should expect an initial Os isotopic signature in the ca. 473 Ma bornite that is equivalent to or more radiogenic (i.e., higher) than the initial Os isotopic composition of the ca. 609 Ma carrolite stage in evaporite breccia (Os_i_ = 3.2 ± 0.9) and the ca. 518–517 Ma stratiform carrolite stage in the Upper and Lower Orebodies (Os_i_ = 3.7 ± 2.4 & 4.8 ± 1.2). Yet, the bornite Os_i_ of 0.4 ± 0.1 suggests that a far more juvenile crustal source of common Os involved during bornite precipitation must be sought.

A possible source of juvenile Os includes the syn-orogenic intrusions responsible for crustal heating during the ca. 570–520 Ma A-type magmatism that produced the Hook Batholith^45^, including its northern, magnetically interpreted subsurface extension (presently located several tens of kilometres to the south of the DRC-Zambia border)^4,46^. Magmatic-hydrothermal systems in connection with granite plutons have been identified as being at the root of multi-stage REE-Y-Co-Cu-Au sediment-hosted mineralisation within large Mesoproterozoic sedimentary basins characterised by large-scale fluxes of evaporitic brines, e.g., the Idaho Cobalt Belt within the Belt-Purcell Basin, USA^47,48^. Therefore, a contribution by the Hook Batholith and its northern subsurface extension as heat engine and source of Os (and possibly other metals such as Cu) for the ca. 473 Ma bornite stage is plausible. Indeed, modelling of crustal fluid flow during collisional overthrust accompanied by magma emplacement has constrained the possibility of long-lived fluid flow up a temperature gradient^49^. The emplacement of magmatic intrusions in surrounding crustal rocks is thus accompanied by large-scale hydrofracturing over tens of kilometres. In such a setting, fluid fluxes produced by magmatism and crustal thickening are substantial and may last up to 35 Myr^49^. To further this hypothesis, the stratiform and veinlet-type carrolite mineralisation in the arenitic dolomite part of the Upper Orebody has disturbed Re-Os systematics that yields an errorchron (MSWD = 21) with a date of 489 ± 59 Ma and an Os_i_ of −0.1 ± 3.2. This arenitic dolomite bears free gold (see Supplementary Figure 1) that might have been sourced from the same granitoid source during this ca. 473 Ma bornite mineralisation stage.

Our approach based on petrographically-constrained Re-Os analyses of mono-mineralic sulphide aliquots and isochron regression, has yielded four mineralisation ages with robust geological and geodynamic consistency, quite dissimilar to the ~600 Myr spread in model ages previously reported for Cu-Co mineralisation from Kamoto^50^. In detail, our study supports the epigenetic introduction of Cu ± Co as disseminated, stratiform, and veinlet-type mineralisation in variably metamorphosed host rocks in the Lufilian fold-and-thrust belt during the later stages of this orogeny between ~540 and 490 Ma^4^. However, our results show that not all mineralisation was formed during this sole 50-Myr Cambrian window^4^ that largely post-dated halokinesis^17,22,23^. Indeed, we have identified two additional and significant mineralisation stages: (1) an epigenetic Co-Cu mineralisation stage replacing evaporite breccia at ca. 609 Ma, thereby placing halokinesis at a minimum age limit coeval with the transition from basinal sedimentation to synorogenic sedimentation (Fig. 3), and (2) an epigenetic Cu-Au mineralisation stage at ca. 473 Ma during orogenic exhumation as a possible far-field effect of large-scale fluid flow and heat transfer triggered by magmatic intrusions in the centre of the orogen. To end with, our study finds no evidence for some early burial diagenetic mineralisation^2,15–18^ that would have occurred prior to rock deformation caused by halokinesis as early as ca. 727 Ma and deposition of the Grand Conglomérat diamictite^17,25^.

### Constraints for the origin of the Central African Copperbelt

The new Re-Os mineralisation ages from Kamoto support the following views and genetic concepts presented for the origin of the Cu ± Co deposits in the Zambian part of the Central African Copperbelt^4,7–9,51^, including the giant deposits at Nchanga^9,51^: (1) strong link between ore formation and the development of structures during basin inversion and the onset of the Lufilian orogeny^4,51^; (2) leaching of Cu (and Co) from basement^4,8,51^; (3) dissolution of evaporites^23,51^; and (4) mineralisation during fold and thrust deformation^51^.

Building on this genetic model for the Zambian part of the Central African Copperbelt, we propose that the remarkable endowment of Cu-Co mineralisation in the Central African Copperbelt as a whole reflects the convergence of specific conditions and processes: (1) a Lufilian fold-and-thrust belt with known linkages to salt tectonics; (2) the efficiency of compressive tectonics during the Lufilian Orogeny on fluid mobilization, overpressuring and saline fluid expulsion for Cu-Co mineralisation; (3) interaction of dense brines with a fertile cupriferous basement, and possibly Neoproterozoic oceanic crust lithologies as a source of cobalt; (4) the possible impact of the demise of the Neoproterozoic snowball events^32^, whereby the penetration of glacial meltwaters caused disequilibrium in basin-scale salinity pattern, which was counterbalanced by enhanced evaporite dissolution with impacts on salt tectonics^20^; and (5) the contribution of this extensive evaporite dissolution onto large-scale, low-velocity convection of high salinity aqueous fluids predicted by numerical modelling and thermodynamic properties of basinal brines^36^.

In light of our data and this proposal for the origin of Cu-Co mineralisation in the Central African Copperbelt, direct and precise geochronological constraints from individual Cu-Co sulphides (carrolite and bornite) should be useful for the understanding of the other controversial Cu-Co deposits in pristine and metamorphosed sedimentary rocks of the Central African Copperbelt, and those worldwide.

## Methods

### Preparation of sulphide mineral separates

A total of 15 carrolite- and/or bornite-mineralised samples from the Upper Orebody, Lower Orebody and evaporitic breccia from the Cu-Co Kamoto deposit, DRC, were processed prior to Re-Os isotope geochemistry (Details of sample characterisation in the Supplementary Data Table). All samples were cut into slabs that were thoroughly cleaned using silicon carbide grit, milli-Q water and ethanol to remove any metal traces left by hammering or sawing. All samples were crushed using a zirconia ceramic dish and puck and sieved through disposable home-made nylon sieves to produce 70‒200 and +70 mesh size fractions. A Frantz Isodynamic Separator was used to produce magnetic (M) and non-magnetic (NM) sub-fractions from the 70‒200 mesh fractions by applying successive 1.1 and 1.7 amp currents for all samples with 15° side slope and 10° forward slope. Bornite ± gangue minerals compose the M.1.1 sub-fractions, whilst carrolite ± gangue minerals were collected in the M1.7 sub-fractions after treatment of the NM.1.1 sub-fractions. The sulphide species were then isolated from remaining gangue minerals into final sulphide mineral separates through heavy liquid separation using Sodium Polytungstate (SPT, specific gravity of 2.86).

### Re-Os isotope geochemistry

For each analysis, between 15 and 750 mg of carrolite or bornite mineral separates was weighed and transferred into a thick-walled borosilicate Carius tube. Each sample was dissolved in inverse Aqua Regia (~3 mL of 11 N HCl and ~6 mL 16 N HNO_3_) with a known amount of ^185^Re+^190^Os spike solution at 210 °C for 24 hours. The full Re-Os laboratory protocol used in the present work is described in full in refs^52–54^. Rhenium (Re) and Os analysis and isotopic compositions were determined by negative thermal ionization mass spectrometry (N-TIMS) using a ThermoScientific Triton mass spectrometer at the Laboratory for source rock and sulphide geochronology and geochemistry, and Arthur Holmes Laboratory in the Durham Geochemistry Centre, Durham University, UK. Rhenium was measured as ReO_4_^−^ in static mode on Faraday collectors, whereas Os was measured as OsO_3_^−^ in peak-hopping mode on SEM with a constant flow of oxygen (refs^54,55^). Measurement quality was monitored by repeated measurements of in-house Re (^185^Re/^187^Re = 0.59892 ± 0.00203, *n* = 74) and Os (“DROsS 4.5b”, ^187^Os/^188^Os = 0.160869 ± 0.000410, *n* = 100) standard solutions. Total procedural blanks for each set of samples are reported in the Supplementary Data Table. All Re-Os ages are reported as Model 1 or Model 3 isochrons through regression in ^187^Os/^188^Os vs. ^187^Re/^188^Os space of the Re-Os data which are reported at the 2σ level (95% level of confidence, *Isoplot* v 4.15 program; ref.^56^).

### Sulphide petrography and quality control of mineral separates

Polished thin sections of the 15 samples were studied by means of transmitted and reflected light microscopy in order to establish, prior to mineral separation, the paragenetic relationships between sulphides and gangue minerals, as well as the relative timing between carrolite and bornite. In addition, an aliquot of each sulphide mineral separate was embedded in epoxy. The mounts were studied by scanning electron microscopy (SEM) using a Hitachi SU-70 FEG SEM operated in backscattered electron mode (SEM-BSE, beam conditions of 20 kV). To further this quality control of mono-mineralic sulphide separates, these qualitative observations were complemented by point wavelength-dispersive spectroscopy (WDS) analyses of carrolite and bornite in the mounts using the following suite of elements: S, Fe, Co, Ni, Cu, Cd, and Te.

## Electronic supplementary material

Supplementary Figure and Table

## Data Availability

All Re-Os isotope data are available in the Supplementary Data Table.
