# Supplementary material for: Sulphide Re-Os geochronology links orogenesis, salt and Cu-Co ores in the Central African Copperbelt
Source: Sci Rep. 2018 Oct 8;8:14946. doi: 10.1038/s41598-018-33399-7 (PMC6175924; doi:10.1038/s41598-018-33399-7)
Supplement: Supplementary file 1 — Supplementary Figure and Table [file 41598_2018_33399_MOESM1_ESM.pdf]

Sulphide Re-Os geochronology links orogenesis, salt and Cu-Co ores in the Central African Copperbelt

Saintilan N.J.[1,*], Selby D.[1,2], Creaser R.A.[3], and Dewaele S.[4,5]

[1]*Department of Earth Sciences, University of Durham, Durham DH1 3LE, United Kingdom*

[2]*State Key Laboratory of Geological Processes and Mineral Resources, School of Earth Resources, China University of Geosciences, Wuhan, China*

[3]*Department of Earth and Atmospheric Sciences, University of Alberta, Edmonton, Alberta, T6G 2E3, Canada*

[4]*Royal Museum for Central Africa, Leuvensesteenweg 13, B-3080 Tervuren, Belgium*

[5]*Mineralogy and Petrology, Department of Geology, Ghent University, Krijgslaan 281 S8, B-9000 Ghent, Belgium*

*Corresponding author:

Saintilan N.J.

njd.saintilan@gmail.com

“Sulphide Re-Os geochronology links orogenesis, salt and Cu-Co ores in the Central African Copperbelt” by Saintilan, N.J., Selby, D., Creaser, R.A., and Dewaele, S.

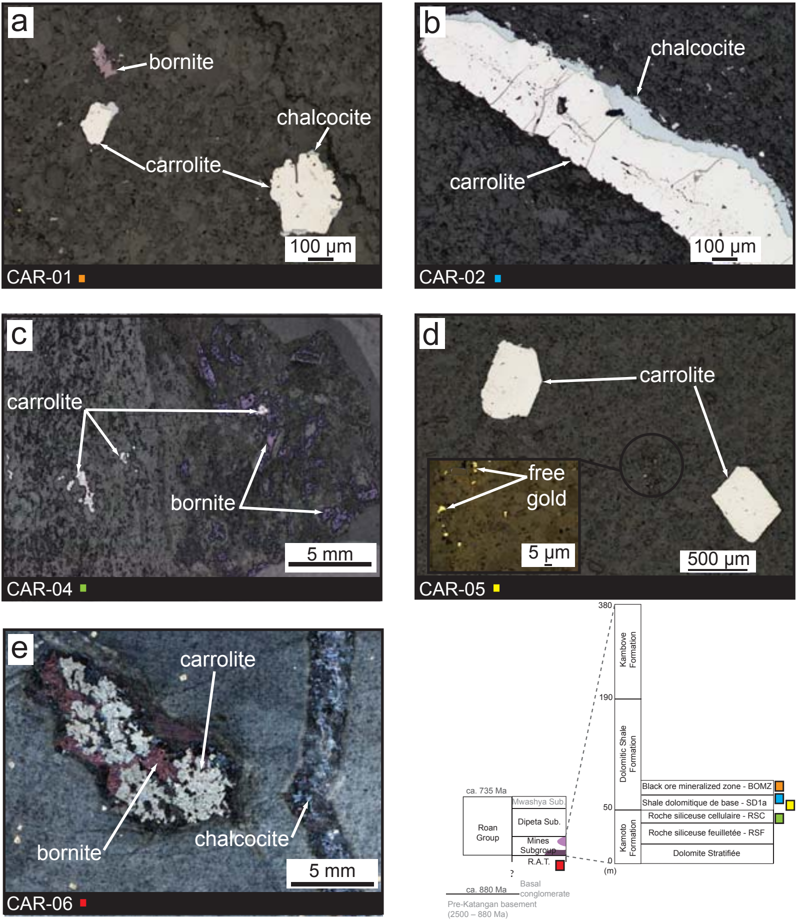

Supplementary Figure 1. Paragenetic relationships between carrolite, bornite and chalcocite in representative samples used in this study. The stratigraphy column shows the position of the samples within the Roan Group. **a.** Reflected light microscopy photograph of carrolite, bornite and chalcocite in this paragenetic order in sample CAR-01 (stratiform Upper Orebody). **b.** Reflected light microscopy photograph of carrolite and chalcocite in sample CAR-02 (stratiform Upper Orebody). **c.** Reflected light microscopy photograph of carrolite and bornite in this paragenetic order in sample CAR-04 (epigenetic sulphide mineralisation in evaporite breccia). **d.** Reflected light microscopy photograph of carrolite in sample CAR-05 (Upper Orebody). The inset shows free gold disseminated in this sample. **e.** Macroscopic scan of a slab of sample CAR-06 (stratiform Lower Orebody) showing carrolite, bornite and chalcocite in this paragenetic order. Chalcocite is mostly present in quartz-chalcocite veins.

Sulphide Re-Os geochronology links orogenesis,salt and Cu-Co ores in the Central African Copperbelt, by Nicolas J. Saintilan, David Selby, Robert A. Creaser, and Stijn Dewaele.

Supplementary Data Table 1. Re-Os isotope geochemistry data for the 15 samples studied from the Kamoto Cu-Co deposit, Democratic Republic of Congo. The lithostratigraphic positions of the samples within the Roan Group are given. The blank compositions of the various runs of Re-Os isotope geochemistry are given. Model ages for the six aliquots from the three CAR-04 samples were calculated with an Osi of 3.2 ± 0.9 Ma. Uncertainty on the model ages are presented as x[y] Ma, with x being the isotopic tracer + analytical uncertainty, and [y] being the isotopic tracer + analytical + decay constant

Batch	Mineral fraction ID	Sample	Stratigraphic position (Stratigraphy after El Desouky et al. 2009)	Aliquot weight (mg)	Re (ppb)	± 2σ	Total Os (ppt)	± 2σ	$^{187}Re/^{188}Os$	± 2σ	$^{187}Os/^{188}Os$	± 2σ	rho	%Re blank	%^{187}Os blank	%^{188}Os blank	Model ages (Ma)
RO873-1	CAR-01 M1.7	CAR-01 sample 1	Mines Subgroup, Dolomitic Shale Formation, Upper	225.00	44.0	0.4	323	2	3853	36	37.5	0.2	0.397	0.02	0.01	0.59	
RO885-4	CAR-01 M1.7	CAR-01 sample 1	Orebody - Black ore mineralized zone	152.03	20.4	0.1	248	1	806	5	8.1	0.0	0.547	0.25	0.08	2.96	Aliquots involved in the ca. 518 Ma Re-Os isochron age
RO930-1	CAR-01 M1.7	CAR-01 sample 2	Mines Subgroup, Dolomitic Shale Formation, Upper	225.87	36.3	0.1	251	36	2549	236	20.6	3.5	0.544	0.03	0.02	0.47	
RO930-3	CAR-01 M1.7	CAR-01 sample 2	Orebody - Shale Dolomitique de base	56.27	55.1	0.2	368	7	5610	88	52.0	0.9	0.900	0.07	0.04	2.69	
RO873-2	CAR-02 M1.7	CAR-02 sample 1	Mines Subgroup, Dolomitic Shale Formation, Upper	334.31	18.0	0.1	143	2	2855	32	28.6	0.3	0.460	0.04	0.02	0.72	
RO873-3	CAR-02 M1.7	CAR-02 sample 1	Orebody - Shale Dolomitique de base	308.08	18.0	0.1	142	1	2875	32	28.7	0.3	0.502	0.04	0.02	0.79	
RO930-4	CAR-02 M1.7	CAR-02 sample 2	Mines Subgroup, Dolomitic Shale Formation, Upper	264.17	17.5	0.1	138	2	2690	27	26.3	0.4	0.581	0.05	0.03	0.88	
RO930-5	CAR-02 M1.7	CAR-02 sample 2	Orebody - Shale Dolomitique de base	49.49	19.2	0.1	137	5	2820	88	24.5	0.9	0.827	0.24	0.15	4.34	
RO885-5	CAR-03 M1.7	CAR-03 sample 1	Mines Subgroup, Dolomitic Shale Formation, Roches	329.68	95.4	0.3	767	4	3023	15	31.1	0.2	0.506	0.02	0.01	1.11	Aliquots involved in the ca. 517 Ma Re-Os isochron
RO892-2	CAR-03 M1.7	CAR-03 sample 1	Siliceuses Cellulaires	16.53	110.1	0.5	928	25	2396	54	24.6	0.7	0.787	0.42	0.13	13.35	
RO930-6	CAR-03 M1.7	CAR-03 sample 2	Mines Subgroup, Dolomitic Shale Formation, Roches Siliceuses Cellulaires	119.92	89.3	0.3	690	4	3484	20	35.3	0.2	0.605	0.02	0.01	0.49	
RO885-7	CAR-04 M1.7	CAR-04 sample 1	Mines Subgroup, Dolomitic Shale Formation,	443.59	139.2	0.5	944	6	18888	96	196	1	0.509	0.01	0.00	3.45	610 ± 7[8]
R0885-8	CAR-04 M1.7	CAR-04 sample 1	Evaporite Breccia in Roches Siliceuses Cellulaires	759.57	151.5	0.5	1010	7	24538	130	253	2	0.485	0.01	0.00	2.43	608 ± 6[7]
RO892-3	CAR-04 M1.7	CAR-04 sample 1		106.54	142.3	0.5	960	17	20577	272	213	3	0.905	0.05	0.02	13.71	610 ± 7 [8]
RO930-7	CAR-04 M1.7	CAR-04 sample 2	Mines Subgroup, Dolomitic Shale Formation, Evaporite Breccia in Roches Siliceuses Cellulaires	125.41	131.6	0.5	915	18	10940	142	114	2	0.741	0.03	0.01	1.98	603 ± 12[13]
RO930-8	CAR-04 M1.7	CAR-04 sample 3	Mines Subgroup, Dolomitic Shale Formation,	62.68	135.2	0.5	951	7	10641	65	111.6	0.7	0.652	0.01	0.00	0.69	608 ± 11[12]
RO930-9	CAR-04 M1.7	CAR-04 sample 3	Evaporite Breccia in Roches Siliceuses Cellulaires	173.58	150.8	0.5	1079	9	9203	58	97.3	0.6	0.667	0.01	0.01	0.74	610 ± 12[13]
RO873-4	CAR-05 M1.7	CAR-05 sample 1	Mines Subgroup, Dolomitic Shale Formation, Upper	280.12	54.4	0.4	350	2	3930	36	32.7	0.2	0.349	0.02	0.01	0.39	Aliquots involved in the ca. 489 Ma Re-Os errorchron
RO873-5	CAR-05 M1.7	CAR-05 sample 1	Orebody - Shale Dolomitique de base	107.95	38.5	0.3	249	3	3413	42	27.6	0.3	0.601	0.06	0.04	1.24	
RO930-11	CAR-05 M1.7	CAR-05 sample 2	Mines Subgroup, Dolomitic Shale Formation, Upper	205.35	8.9	0.0	90	1	948	8	7.7	0.1	0.472	0.13	0.08	0.79	
RO930-12	CAR-05 M1.7	CAR-05 sample 2 (vein)	Orebody - Shale Dolomitique de base	243.46	25.2	0.1	159	1	3821	27	30.7	0.2	0.720	0.04	0.02	0.94	
RO934-4	CAR-07 M1.7	CAR-07 sample 1	Mines Subgroup, Kamoto Formation, Lower Orebody - Roches Siliceuses Feuilletées	406.08	31.7	0.1	473	2	830	5	12.2	0.1	0.561	0.02	0.01	0.10	Aliquots involved in the ca. 517 Ma Re-Os isochron age
RO934-5	CAR-07 M1.7	CAR-07 sample 2	Mines Subgroup, Kamoto Formation, Lower Orebody - Roches Siliceuses Feuilletées	421.99	17.8	0.1	257	1	863	5	12.3	0.1	0.573	0.03	0.01	0.18	
RO934-6	CAR-08 M1.7	CAR-08 sample 1	Mines Subgroup, Kamoto Formation, Lower Orebody - Roches Siliceuses Feuilletées	135.06	9.5	0.0	102	2	1459	21	17.4	0.3	0.768	0.18	0.08	1.71	
RO930-2	Bn-01 M1.1	CAR-01 sample 1	Mines Subgroup, Dolomitic Shale Formation, Upper Orebody - Black ore mineralized zone	122.58	2.4	0.0	75	3	194	16	2.0	0.2	0.708	0.77	0.40	0.99	Aliquots involved in the ca. 473 Ma Re-Os isochron age
RO892-1	Bn-04 M1.1	CAR-04 sample 1	Mines Subgroup, Dolomitic Shale Formation, Evaporite Breccia in Roches Siliceuses Cellulaires	65.82	27.0	0.1	208	4	1836	33	14.9	0.3	0.775	0.43	0.17	10.78	
RO934-2	Bn-06 M1.1	CAR-06 sample 1	Mines Subgroup, Kamoto Formation, Lower Orebody -	390.90	4.3	0.0	25	1	5918	171	48	1	0.947	0.14	0.09	5.13	
RO934-3	Bn-06 M1.1	CAR-06 sample 1	Roches Argilo Talqueuses Grises	425.80	4.6	0.0	26	1	6557	177	52	1	0.943	0.12	0.08	4.82	
RO934-8	Bn-06 M1.1	CAR-06 sample 1		401.25	3.9	0.0	22	1	6265	204	50	2	0.952	0.15	0.10	5.82	

Blank series	Batch	Os (ppt)	± 2σ	$^{187}Os/^{188}Os$	± 2σ	Re (ppt)	± 2σ
Series 1	RO875, RO893, RO892	0.57	0.02	0.21	0.06	7.68	1.34
Series 2	RO930, RO934	0.08	0.02	0.80	0.06	2.30	0.18
